# Widening the Perspectives for Legume Consumption: The Case of Bioactive Non-nutrients

**DOI:** 10.3389/fpls.2022.772054

**Published:** 2022-02-10

**Authors:** Rafaela Geraldo, Carla S. Santos, Elisabete Pinto, Marta W. Vasconcelos

**Affiliations:** CBQF - Centro de Biotecnologia e Química Fina – Laboratório Associado, Escola Superior de Biotecnologia, Universidade Católica Portuguesa, Porto, Portugal

**Keywords:** anti-nutrients, bioactive, legume grains, health, sustainability

## Abstract

Legume grains have provided essential nutrients in human diets for centuries, being excellent sources of proteins, carbohydrates, fatty acids, and fibers. They also contain several non-nutrients that historically have been connotated as toxic but that in recent years have been shown to have interesting bioactive properties. The discussion on the role of bioactive non-nutrients is becoming more important due to increasing science-based evidence on their potential antioxidant, hypoglycemic, hypolipidemic, and anticarcinogenic properties. At a time when legume-based products consumption is being strongly promoted by national governments and health authorities, there is a need to clearly define the recommended levels of such non-nutrients in human diets. However, there is insufficient data determining the ideal amount of non-nutrients in legume grains, which will exert the most positive health benefits. This is aligned with insufficient studies that clearly demonstrate if the positive health effects are due to the presence of specific non-nutrients or a result of a dietary balance. In fact, rather than looking directly at the individual food components, most nutritional epidemiology studies relate disease risk with the food and dietary patterns. The purpose of this perspective paper is to explore different types of non-nutrients present in legume grains, discuss the current evidence on their health benefits, and provide awareness for the need for more studies to define a recommended amount of each compound to identify the best approaches, either to enhance or reduce their levels.

## Introduction

The intensification of agriculture and the unbalanced consumption of animal protein has called for increased consumption of alternative sources of protein, such as legumes. However, legume production and consumption levels are at a historic low in many parts of the world, including in many European countries (Cusworth et al., 2021). Food and feed-wise, legumes are often subdivided into three subgroups: fresh legumes (e.g., beans and peas), oilseed legumes (like peanuts and soybeans), and pulses (dried and edible seeds of legume plants, such as chickpeas, dried peas, and dried beans; Mullins and Arjmandi, 2021). Legume production may help reduce greenhouse gas emissions, improve soil carbon sequestration, and overall reduce fossil energy inputs in farming systems (Mus et al., 2016; Stagnari et al., 2017). Oftentimes legumes grow well in poor soils and with unfavorable weather conditions and may be used as cover crops, which contribute to a reduction in soil erosion. These benefits, combined with the fact that they form symbiotic relations with nitrogen-fixing bacteria, make them excellent rotational crops (Maphosa and Jideani, 2017; Liu et al., 2018).

Legumes are generally low in fat, cholesterol-free and excellent sources of protein/amino acids, providing a large share of human dietary protein requirement (Smýkal et al., 2015), fatty acids, fibers, carbohydrates, vitamins, and minerals (Ganesan and Xu, 2017; Mirali et al., 2017; Bazghaleh et al., 2018; Balázs et al., 2021; Iannetta et al., 2021), like calcium (Ca), chromium (Cr), copper (Cu), iron (Fe), magnesium (Mg), phosphorus (P), potassium (K), selenium (Se), and zinc (Zn; Kouris-Blazos and Belski, 2016).

The consumption of legumes, as a part of a balanced diet, can bring human health benefits, including a reduced risk of cardiovascular disease (CVD; Marventano et al., 2017) and related CVD issues, like obesity, high blood pressure, type-2 diabetes, dyslipidemia, and stroke (Polak et al., 2015; Becerra-Tomás et al., 2019; Ferreira et al., 2021; Mullins and Arjmandi, 2021). Its reduction is possible due to the low glycemic index of legumes (avoid peaks in blood glucose), their high fiber content, and the presence of the non-nutrients (phytosterols, saponins, and lectins, among others; Duranti, 2006). Besides, legumes also improve the microbial diversity of gut, colon health, oxidative stress, inflammatory status, and even help to reduce cancer (Santos et al., 2017; Mirmiran et al., 2018; Mullins and Arjmandi, 2021; Ferreira et al., 2022).

Nonetheless, legumes have historically been associated with the presence of specific classes of anti-nutrients (or bioactive non-nutrients) which, if processed inappropriately, may have secondary effects, such as toxicity or legume-related food allergies (e.g., peanut and soybean). The negative connotation began several years ago, and one of the earliest pieces of evidence comes from the story of the Greek philosopher and mathematician, Pythagoras, who forbade his disciples to consume the Greek fava beans because it made many people sick with the so-called “favism” (Meletis, 2012). It is claimed that Pythagoras died at the hands of the enemy because he decided not to escape through a fava bean field (Meletis, 2012). Currently, it is known that favism is a form of hemolytic anemia and jaundice caused by a genetically inherited deficiency in the enzyme glucose-6-phosphate dehydrogenase (G6PD; Luzzatto and Arese, 2018). The cause of favism in such individuals is due to the presence of two fava bean anti-nutrients, the pyrimidine glycosides vicine and convicine (Luzzatto and Arese, 2018; Khazaei et al., 2019). These compounds are thermostable, but their concentration can be greatly reduced by seed soaking, frying, boiling, microwave irradiation, roasting, or fermenting (Pulkkinen et al., 2019). For non-nutrients that are clearly unsafe, breeding could help in the reduction/elimination of undesired non-nutrients levels (Khazaei et al., 2019; Robinson et al., 2019). In fact, low vicine and convicine fava bean cultivars are now available and researchers are also investigating ways to completely eliminate them (Khazaei et al., 2019). While the reduction of vicine and convicine levels has been successfully achieved, with a consensus that this reduction would be important for a broader consumption of fava beans, we cannot say the same for all anti-nutritional compounds. For some, health benefits may be promoted, and increasing their levels could be considered. Nonetheless, a thorough discussion is needed to decide when (and if) these compounds should be bred “in” or “out” or kept “as is.”

Even though legumes provide several health and environmental advantages there is a persistent barrier to their increased consumption related to the presence of bioactive non-nutrients. For example, legumes are highly associated with causes of unwanted flatulence, due to the presence of oligosaccharides (raffinose, stachyose, and verbascose; Abdel-Gawad, 1993; Han and Baik, 2006). In times where legumes have been put forward as an important protein source and as a vehicle to provide well-balanced nutrition, while safeguarding the environment, there is a need to clarify the real concerns (or lack thereof) of these compounds. Does the presence of non-nutrients bring positive or negative impacts, and how to balance the two? The present perspective takes a close look at this question and discusses some of the angles that need to be considered when discussing future research needs.

## Non-nutrients

The non-nutrients can be broadly divided into two major categories: the proteinaceous group and the non-proteinaceous group. The former includes lectins, agglutinins, bioactive peptides, and protease inhibitors, and the second group includes alkaloids, phytic acid, tannins, and saponins (Sánchez-Chino et al., 2015). The accumulation in edible seeds is a natural process, triggered by plant defense mechanisms against insects, parasites, fungi, and herbivorous animals (Sánchez-Chino et al., 2015). They can also act as a nutritional pool to maintain plant growth under unfavorable conditions (Sánchez-Chino et al., 2015). Although some non-nutrients are mostly found in certain types of legumes, such as vicine and convicine in fava bean (Khamassi et al., 2013), not all of them are legume-exclusive; phytic acid is also present in cereals, oil seeds, nuts, and plants (Gupta et al., 2015); oxalates in spinach, Swiss chard, rhubarb, and potatoes; tannins in tea, cocoa, grapes, and wine (Petroski and Minich, 2020).

**Figure 1:**
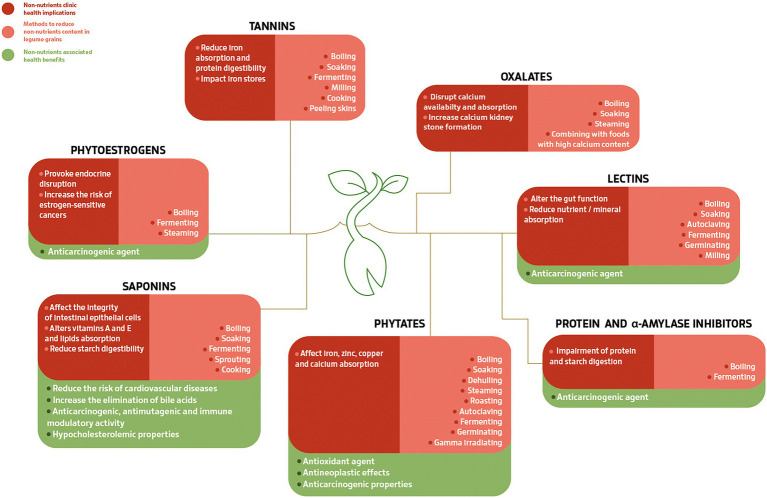
Different non-nutrients found in legume grains, their clinical health implications, and methods to reduce their content.

It is important to note that legumes that share similar nutritional profiles may have significant variations in the relative abundances of individual nutrients (Mirali et al., 2017). This variability extends not only to protein and other macronutrients but also for bioactive compounds (Table 1), and this should be considered when evaluating the right amount of each in a dietary serving. Among the proteinaceous non-nutrients, the glycoproteins lectins or hemagglutinins have the capacity of reversibly attaching carbohydrates on cells, like red blood cells, resulting in erythrocyte agglutination (Petroski and Minich, 2020; Samtiya et al., 2020). Lectins, present especially in common beans (*Phaseolus vulgaris*) and peas (*Pisum sativum*; Table 1), have a negative role in nutrient absorption (by binding intestinal epithelial cells), and in the integrity of the mucosa, causing intestinal hyperplasia and high permeability (Figure 1; Petroski and Minich, 2020; Samtiya et al., 2020), which allow bacteria contact with the bloodstream (Samtiya et al., 2020). Despite lectins are resistant to enzymes in the gastrointestinal tract, they can be reduced/removed by boiling, soaking, autoclaving, fermenting, germinating, and milling (Figure 1; Petroski and Minich, 2020). For example, boiling white and red kidney beans can eliminate lectin content (Nciri et al., 2015). However, lectins may have clinical benefits, for example, some studies show that they can recognize different glycan production of cancer cells and therefore can be potentially used in cancer treatments (Figure 1; Panda et al., 2014; Gautam et al., 2018, 2020; Bhutia et al., 2019; Mullins and Arjmandi, 2021). Besides, they positively activate the immune system, modifying the expression of interleukins and some protein kinases, and have been demonstrated as possible antiviral and antimicrobial agents (Figure 1; Lagarda-Diaz et al., 2017; Mullins and Arjmandi, 2021). For instance, in the treatment of severe acute respiratory syndrome coronavirus 2 (SARS-CoV-2), responsible for the currently COVID-19 pandemic, lectins can bind complex-type-N-glycans on viral glycoproteins, like coronaviruses spike and prevent the production of viral proteins and the cytopathic effect in host cells (Liu et al., 2020).

**Table 1:** Summary of main legume species and concentrations of the non-nutrient’s lectins, oxalates, total phenolics, phytates, saponins, and tannins (in yield range), and of trypsin and alpha-amylase inhibitors (in activity units).

Non-nutrient	Legume species	Yield range (mg/100 g seeds)	References
Lectins	*Cicer arietinum* L.	95	Gautam et al., 2018
	*Glycine max*	360	Barca et al., 1991
	*Lens culinaris*	48	El-Araby et al., 2020
	*Phaseolus vulgaris*	13–1,100 174	Lam and Ng, 2010 Shang et al., 2016
	*Pisum sativum*	148–160	El-Araby et al., 2020
	*Vicia faba*	50	El-Araby et al., 2020
Oxalates	*Arachis hypogaea*	41	Guo et al., 2021
	*Cicer arietinum*	192–199	Shi et al., 2018
	*Glycine max*	370	
	*Lens culinaris*	168–289	
	*Phaseolus vulgaris*	99–117	
	*Pisum sativum*	244–280	
	*Macrotyloma uniflorum*	88–123	Vashishth et al., 2021
	*Vicia faba*	241–291	Shi et al., 2018
Total phenolics	*Canavalia* spp.	640–1,818	Sridhar and Sahadevan, 2006
	*Glycine max*	1.77–2.48	Król-Grzymała and Amarowicz, 2020
	*Lens culinaris*	12	Piecyk et al., 2012
	*Lupinus angustifolius*	94.66	Karnpanit et al., 2016
	*Mucuna pruriens*	0.565	Siddhuraju and Becker, 2005
	*Phaseolus vulgaris*	35.5–45.6 105.8	Barreto et al., 2021 Piecyk et al., 2012
	*Pisum sativum*	11.6	Piecyk et al., 2012
	*Vicia faba*		
	*Vigna unguiculata*	1,210	Kalpanadevi and Mohan, 2013
Phytates	*Cicer arietinum* L.	1,133–1,400	Shi et al., 2018
	*Glycine max*	2,291	
	*Lens culinaris*	856–1710	
	*Lupinus angustifolius*	0.80	Karnpanit et al., 2016
	*Macrotyloma uniflorum*	42–45	Vashishth et al., 2021
	*Mucuna pruriens*	950	Siddhuraju and Becker, 2005
	*Phaseolus vulgaris*	310 1,580 1,564–1,882 1,760–2,080	Shang et al., 2016 Carbas et al., 2020 Shi et al., 2018 Barreto et al., 2021
	*Pisum sativum*	855–993	Shi et al., 2018
	*Vicia faba*	1,965 112–1,281	Shi et al., 2018 Mayer Labba et al., 2021
	*Vigna unguiculata*	360–510	Avanza et al., 2013
Saponins	*Cajanus cajan*	2,164	Duhan et al., 2001
	*Canavalia* spp.	571–1,005	Sridhar and Sahadevan, 2006
	*Medicago sativa*	800–1,650	Hadidi et al., 2020
	*Mucuna pruriens*	1,210	Siddhuraju and Becker, 2005
	*Phaseolus vulgaris*	940–1,180 373	Emire and Rakshit, 2007 Shang et al., 2016
	*Vigna radiata*	2,848	Kataria et al., 1988
	*Vigna umbellata*	2,175–2,450	Kaur and Kapoor, 1992
Tannins	*Canavalia* spp.	230–900	Sridhar and Sahadevan, 2006
	*Lupinus angustifolius*	46.41	Karnpanit et al., 2016
	*Macrotyloma uniflorum*	90–92	Vashishth et al., 2021
	*Mucuna pruriens*	300	Siddhuraju and Becker, 2005
	*Phaseolus vulgaris*	170–1,770	Carbas et al., 2020
	*Vicia faba*	1,370	Sharma and Sehgal, 1992
	*Vigna unguiculata*	380 110–820	Kalpanadevi and Mohan, 2013 Avanza et al., 2013
		**Activity units (U/mg)**	
Trypsin inhibitors	*Arachis hypogea*	5.60	Embaby, 2010
	*Cajanus cajan*	4.75	Sangronis and Machado, 2007
	*Cicer arietinum*	12.60–14.51 14.22–16.24	Muzquiz et al., 2012 Shi et al., 2017
	*Lens culinaris*	3–8 7.40 4.98–6.29	Guillamón et al., 2008 Świeca and Baraniak, 2014 Shi et al., 2017
	*Phaseolus vulgaris*	17–51 15.18–20.83	Guillamón et al., 2008 Shi et al., 2017
	*Pisum sativum*	5.75–12.55 3.16–4.92	Muzquiz et al., 2012 Shi et al., 2017
	*Vicia faba*	5–10 5.96–6.10 4.47	Guillamón et al., 2008 Shi et al., 2017 Alonso et al., 2000
	*Vigna unguiculata*	7.52	Rivas-Vega et al., 2006
Alfa-amylase inhibitors	*Cajanus cajan*	0.07	Choi et al., 2019
	*Cicer arietinum*	0.09 0.02–0.08	Choi et al., 2019 Mulimani et al., 1994
	*Phaseolus vulgaris*	0.786–1.37 0.25	Shi et al., 2017 Alonso et al., 2000
	*Vicia faba*	0.02	Alonso et al., 2000
	*Vigna angularis*	0.12	Choi et al., 2019
	*Vigna radiata*	0.14	
	*Vigna unguiculata*	0.18	

Protein and α-amylase inhibitors may present higher activity units in common beans and chickpeas (*Cicer arietinum*; Table 1) and are natural plant inhibitors that interfere with mineral bioavailability, nutrient absorption, and protein and starch digestibility (Figure 1; Sánchez-Chino et al., 2015; Samtiya et al., 2020). Although studies are limited and not recent and this subject remains controversial, it has been broadly reviewed that some of the inhibitors, like Bowman-Birk, may present anticarcinogenic effects (Figure 1; Muzquiz et al., 2012; Sánchez-Chino et al., 2015; Srikanth and Chen, 2016; Kårlund et al., 2021). Boiling and fermenting may reduce their amount (Figure 1; Maphosa and Jideani, 2017), and there are already studies aimed to reduce these compounds, through natural or induced biodiversity screening (Sparvoli et al., 2016).

Oxalates are often labeled as deleterious non-nutrients and are frequently present in soybean, fava bean, and peas (Figure 1), but also in non-legumes (Mitchell et al., 2019; Petroski and Minich, 2020). They are usually associated with a reduction in mineral bioavailability and absorption (through chelating minerals) and with favoring kidney stones formation (Shi et al., 2018; Petroski and Minich, 2020). Oxalates are usually excreted in urine (Shi et al., 2018), and its excretion can be promoted *via* proper hydration, Ca consumption (Ca binds to oxalates during digestion), and vitamin C balance (which may influence the oxalate endogenous production; Mitchell et al., 2019). Boiling, soaking, steaming, and combining with high Ca-rich foods help to reduce oxalate content (Figure 1; Petroski and Minich, 2020). For example, soaking seeds of different legumes species reduced the oxalate content by 17–52% and the reduction even increased after cooking, 31–66% (Shi et al., 2018). Nevertheless, it is necessary to have into account that legumes are not the only oxalate source; cooked and raw spinach is considered the major supplier since ingestion of 50–100 g of spinach (normal portion) provides around 500–1,000 mg of oxalate (Mitchell et al., 2019); also in cocoa powder, oxalates content was found to be 619 mg/100 g; in sweet potatoes 496 mg/100 g and in okra 317 mg/100 g (Siener et al., 2020).

Phytate or phytic acid, a non-proteinaceous non-nutrient (Raes et al., 2014), frequently present in soybeans, fava beans, and common beans (Table 1), can chelate Fe, Zn, and Cu, and can negatively affect their absorption in the gastrointestinal tract (Figure 1; Samtiya et al., 2020). People that consume a large amount of legume grains as a part of their diet can have lower levels of Fe. In extreme cases, this can cause anemia (Shi et al., 2018), if the recommended daily doses are exceeded or it is not maintained a balanced diet. The adequate provision of vitamin C in the diet is a good option to counteract these negative effects since it keeps Fe available for absorption (Bohn et al., 2008; Petroski and Minich, 2020). Nevertheless, phytates have important health benefits, such as anticarcinogenic properties and antioxidant activity (Figure 1), chelating toxic metals, palladium and cadmium, or excess Fe, thus preventing harmful Fenton reactions (Shi et al., 2018; Petroski and Minich, 2020). Several techniques can reduce their content, for example, boiling, autoclaving, among others (Figure 1; Maphosa and Jideani, 2017) and, in the last decades, several mutants with low phytic acid have been developed, like in common beans (Campion et al., 2009; Sparvoli et al., 2016; Cominelli et al., 2018), to improve the nutritional quality of this seed crop (Cominelli et al., 2020).

Phenolic compounds, present in *Canavalia* spp. and cowpea (*Vigna unguiculata*; Table 1), can have anti-inflammatory and antioxidant properties, improve gut health (Filosa et al., 2018), lead to the inhibition of glucose regulation enzymes α-amylase and amyloglucosidase (Sánchez-Chino et al., 2015), and reduce the risk of CVD, type 2 diabetes, metabolic syndrome, ischemic stroke, and atherosclerotic vascular disease (Petroski and Minich, 2020). Nevertheless, not all polyphenolic compounds have health benefits, for example, tannins. Found mostly in the outer layers of grains and seed coats and, in higher concentration, in fava beans (Table 1), but also in cocoa beans, tea, wine, and fruits, they have the capacity of interfering with Fe absorption and storage, contributing to Fe deficiency anemia (Figure 1; Raes et al., 2014; Petroski and Minich, 2020). They can also form protein complexes, reducing protein digestibility and inactivating digestive enzymes (Figure 1; Samtiya et al., 2020). Methods like boiling, soaking, fermenting, milling, cooking, and de-coating allow the reduction of their content in legume seeds (Figure 1; Petroski and Minich, 2020; Samtiya et al., 2020). In the case of fava bean, genetic improvement has been applied to obtain zero-tannin cultivars (Gutierrez et al., 2008). Phytoestrogens, present especially in soy products (tofu, tempeh, and soymilk), have a similar structure to the female primary sex hormone, 17-β-estradiol, and also have some health concerns; they may be involved in endocrine disruption and increase the risk of estrogen-sensitive cancers (Figure 1; Petroski and Minich, 2020). However, there are some references to anticarcinogenic effects (Figure 1; Sánchez-Chino et al., 2015; Petroski and Minich, 2020). These compounds can be reduced through boiling, fermenting, and steaming (Figure 1; Petroski and Minich, 2020).

Saponins in plant foods can interact with erythrocytes increasing the risk of hemolysis, inhibit digestive enzyme activities causing indigestibility disorders, and reduce vitamin absorption (Figure 1; Samtiya et al., 2020). However, saponins can also reduce the risk of CVD, cancer, blood cholesterol, and blood glucose; increase bile acids excretion, cell proliferation regulation, and have anti-inflammatory and immune-stimulatory activities (Figure 1; Sánchez-Chino et al., 2015; Singh et al., 2017). Once again, several standard processing methods are effective at reducing their amount (Figure 1; Maphosa and Jideani, 2017; Samtiya et al., 2020), for example, soaking navy beans reduced the level of saponins by 6.3% and soaking and cooking by 42.3% (Shi et al., 2009).

Since the consumption of non-nutrients has contrasting health effects, the possibility of reducing or increasing their content in different legumes has been considered (Gutierrez et al., 2008; Cominelli et al., 2018; Khazaei et al., 2019). The vast majority can be reduced or even eliminated by traditional food preparation procedures (Figure 1), and proper processing methods can reduce their amount and increase the protein digestibility and biological value of legumes (Samtiya et al., 2020). These methods are well documented in the literature according to the perspective that these compounds need to be eliminated (Samtiya et al., 2020) but the discovery that these can have beneficial effects has opened a new path of study. Some can indeed be present after food preparation procedures, and their health implications need to be further explored. However, benefits or deleterious effects are related to intake amount (Conti et al., 2021), which is absent in the literature, emphasizing the need to develop guidelines for recommended intake. Nevertheless, legumes are currently being used in alternative ways (e.g., flours), where they may not be subjected to these kinds of processing methods, therefore new breeding approaches are required. Hence, further studies on specific levels for these compounds that may bring positive health outcomes without jeopardizing human and animal health are necessary.

Furthermore, climatic changes can have an impact on the composition of these compounds, and thus, the future breeding programs and selection of high or low-bioactive legumes must be adapted (Hummel et al., 2018; Herrera et al., 2019). For example, mild hydric stress in common bean culture increased the non-nutrient content (phenolic compounds and saponins; Herrera et al., 2019).

To better evaluate the real need of reducing non-nutrients levels in plant foods or showing the benefits of such compounds, specific nutritional epidemiology studies are needed, but they are quite limited. It is imperative to have studies looking for associations between foods or even dietary patterns and diseases risk, rather than looking directly at the nutrients and components of individual foods (Hu, 2002). For example, several research studies show an inverse relationship between consumption of different legumes and CVD risk (Macarulla et al., 2001; Jukema et al., 2005; Winham and Hutchins, 2007; Abeysekara et al., 2012; Zhu et al., 2012; Ferreira et al., 2021). This benefit could be partially justified by these bioactive compounds in combination with others, in synergistic relationships (Hu, 2002; Bhupathiraju and Tucker, 2011).

Furthermore, it should be considered that although some non-nutrients are more abundant in specific legumes, their intake dosage, within a diversified diet, can balance the beneficial and adverse effects. This could ensure their recognition as non-nutrient or pro-nutrient (Muzquiz et al., 2012; Popova and Mihaylova, 2019). For example, phytic acid represents a non-nutrient factor in the context of a poor diet, that lacks in minerals and vitamins, or in unfavored segments of the population (such as elders and infants), while it can have health properties in a rich diet, typical of the industrialized countries (Nissar et al., 2017). Saponins may also have opposite effects, that is, when consumed in low amounts may contribute with the previously mentioned benefits, but when ingested in high amounts may have deleterious effects (Kumar and Pandey, 2020). Nonetheless, more studies are needed to determine the recommended amount of these compounds to avoid these harmful effects.

## Future Research Needs

Legume consumption provides health and environmental gains. However, the presence of non-nutrients continues to affect their consumption, and the goal of increasing the levels of these is a complex subject. The purpose of this perspective is not to give breeding directions for these non-nutrients but to raise awareness of this topic and underline the need for further studies and knowledge on specific amounts of these compounds that may bring health benefits without compromising general health and determine the need to either increase or decrease them. These may be a challenge since these compounds are not ingested isolated but in meals containing further compounds that can have synergic relationships. Besides, the human clinical trials that investigate the non-nutrients effects are quite limited and the alternative epidemiological/observational studies used are difficult to implement due to different variables. There are also great discrepancies in legume consumption habits, linked to cultural aspects, dietary habits, processing methods, and socioeconomics, among others, that need to be integrated into a multidisciplinary approach for proper guidance of future research efforts. Therefore, in the future, more research is needed to make a proper position and clarify these knowledge gaps, including a technical perspective from breeders, public health specialists, sociologists, policymakers that takes into consideration all these aspects.

## Data Availability Statement

The raw data supporting the conclusions of this article will be made available by the authors, without undue reservation.

## Author Contributions

MV defined the concept. RG, MV, CS, and EP offered contributions to the design and writing of the manuscript, as well as to the analysis and interpretation of data for the work, and revised the manuscript critically. All authors contributed to the article and approved the submitted version.

## Funding

This research was supported by the European Union’s Horizon 2020 Research and Innovation Programme through project “Realising Dynamic Value Chains for Underutilised Crops” (RADIANT), Grant Agreement number 101000622, and by the Fundação para a Ciência e Tecnologia (FCT, Portugal) through PhD scholarship 2021.05683.BD.

## Conflict of Interest

The authors declare that the research was conducted in the absence of any commercial or financial relationships that could be construed as a potential conflict of interest.

## Publisher’s Note

All claims expressed in this article are solely those of the authors and do not necessarily represent those of their affiliated organizations, or those of the publisher, the editors and the reviewers. Any product that may be evaluated in this article, or claim that may be made by its manufacturer, is not guaranteed or endorsed by the publisher.
